# Perioperative sleep management in cardiac surgery: an evidence-oriented narrative review of pharmacological, behavioral, and respiratory support strategies

**DOI:** 10.3389/fcvm.2026.1853937

**Published:** 2026-07-09

**Authors:** Bo Kong, Yan Yang, Min Song, Meng Yan, Yanhai Meng

**Affiliations:** Department of Adult Cardiac Surgery, Fuwai Hospital, Chinese Academy of Medical Sciences and Peking Union Medical College, Beijing, China

**Keywords:** behavioral intervention, cardiac surgery, perioperative period, pharmacotherapy, respiratory support, sleep disturbance

## Abstract

Perioperative sleep disturbance is a common problem in patients undergoing cardiac surgery. It may manifest as reduced sleep quality, impaired sleep continuity, and disrupted sleep architecture, and may be associated with postoperative delirium, cognitive decline, and delayed recovery. As an evidence-oriented narrative review, this article summarizes pharmacological interventions, behavioral therapies, and respiratory support strategies for perioperative sleep management in cardiac surgery. Current evidence suggests that melatonin and its receptor agonists may help regulate circadian rhythms and improve subjective sleep quality, but their effects on outcomes such as delirium, length of hospital stay, and objective sleep architecture remain inconsistent. Dexmedetomidine has sedative properties resembling non-rapid eye movement sleep; however, findings across studies are inconsistent, and hemodynamic adverse effects require careful attention. Although GABAergic hypnotics may shorten sleep latency, they may increase the risk of respiratory depression, delirium, and adverse cognitive events. Behavioral interventions and environmental optimization are generally feasible, but much of the supporting evidence comes from patients with chronic insomnia, general surgical populations, or ICU settings; therefore, extrapolation to perioperative cardiac surgical patients should be cautious. CPAP, BiPAP, and high-flow nasal cannula oxygen therapy mainly improve oxygenation and reduce respiratory support requirements, whereas their direct sleep-related benefits remain unclear. Overall, risk-stratified multimodal sleep management is clinically rational, but large-scale, multicenter studies are still needed to further clarify its efficacy, safety, and applicable patient populations.

## 1 Introduction

**Figure 1:**
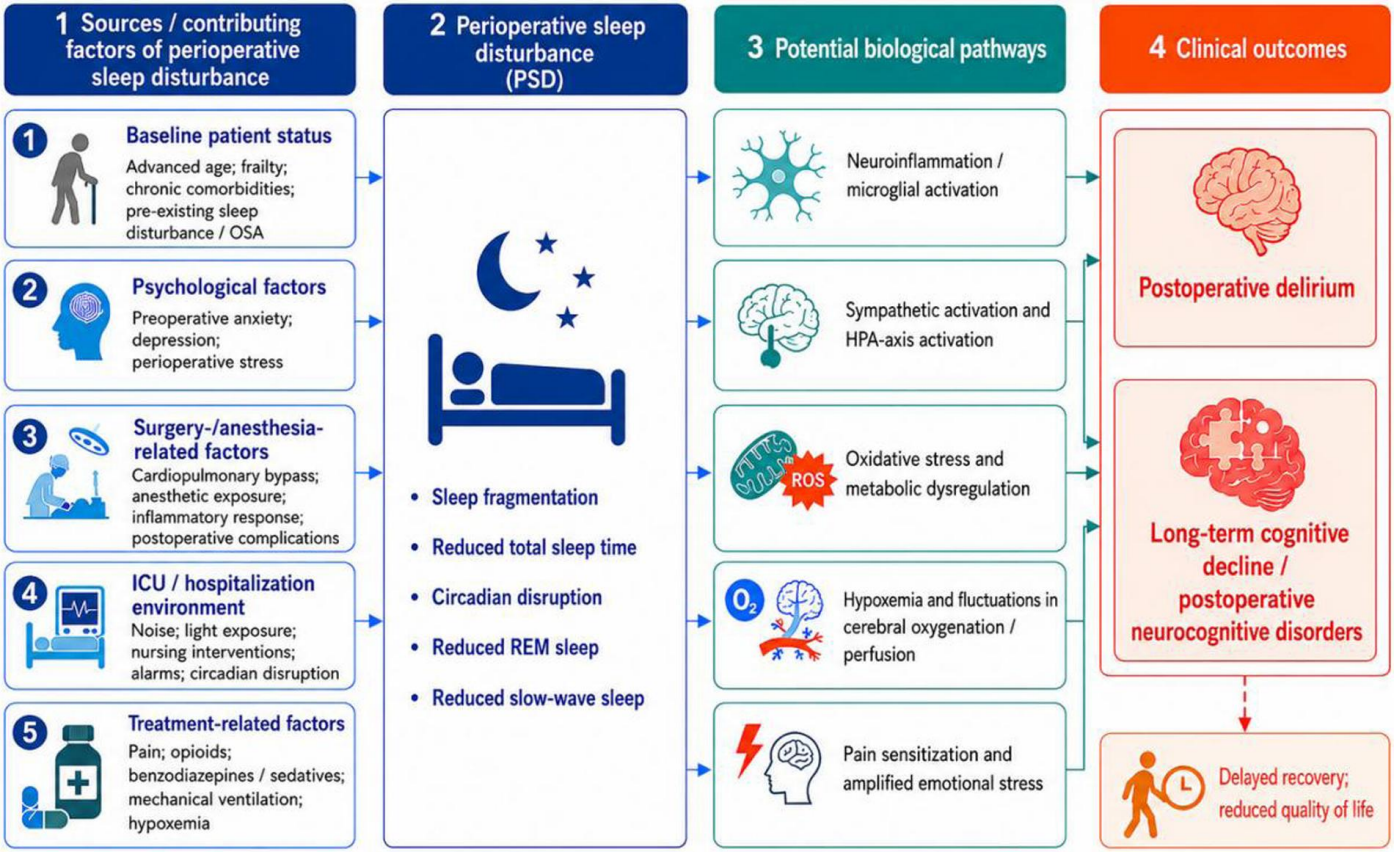
Schematic diagram of the proposed pathways by which PSD may contribute to delirium and long-term cognitive decline.

Perioperative sleep disturbance (PSD) in cardiac surgery refers to reduced sleep quality, difficulty initiating or maintaining sleep, and disrupted sleep architecture during the perioperative period, influenced by psychological, surgical, anesthetic, and environmental factors (1). Cardiac surgical patients are typically older, have a higher burden of comorbidities, and often experience prolonged hospitalization and postoperative intensive care unit (ICU) monitoring. Consequently, their sleep continuity and circadian rhythms are particularly vulnerable to perioperative environmental factors, pain, therapeutic procedures, and psychological stress (2, 3). As a common perioperative problem, PSD is frequently associated with physical discomfort, psychological distress, impaired neurocognitive function, and prolonged recovery (1, 4). Existing studies suggest that cardiac surgical patients may experience varying degrees of sleep disturbance during hospitalization and the postoperative recovery period, which may be related to the quality of postoperative recovery, neurocognitive status, and the complexity of perioperative management (5, 6). PSD may contribute to an increased risk of postoperative delirium and cognitive dysfunction, heightened pain sensitivity, and delayed recovery through mechanisms involving neuroinflammatory activation, alterations in brain structure, and metabolic dysregulation (1, 4). These potential mechanisms may be more pronounced in older patients with chronic diseases and frailty, thereby affecting postoperative rehabilitation and long-term quality of life (Figure 1) (7). Pain, anxiety, and postoperative complications often exhibit complex bidirectional relationships with sleep disturbance and may collectively affect patients’ analgesic requirements, emotional status, and sleep recovery (8, 9). Moreover, reliance solely on commonly used sedative and analgesic agents, such as opioids and benzodiazepines, fails to fundamentally alleviate PSD or mitigate its associated prognostic risks, and certain agents may even increase the incidence of cognitive impairment and persistent sleep disorders (10, 11). Therefore, the management of perioperative sleep disturbance in cardiac surgical patients urgently requires the systematic integration of multiple intervention strategies, including pharmacological, behavioral, and respiratory support approaches. Given the differences in the implementation windows of different interventions, preoperative sleep management in this review includes screening and initiation of interventions in preoperative outpatient clinics or during the pre-admission preparation phase, as well as short-term management after hospital admission and before surgery. This review summarizes and discusses three major strategies for perioperative sleep management in cardiac surgery, namely pharmacological interventions, behavioral therapy, and respiratory support, with the aim of providing a reference for optimizing perioperative sleep management, improving postoperative outcomes, and enhancing patients’ quality of life.

The figure illustrates potential contributors to perioperative sleep disturbance (PSD), associated alterations in sleep patterns, and putative biological pathways that may mediate adverse postoperative neurocognitive outcomes. These pathways should be interpreted as evidence-informed hypotheses rather than definitively proven mechanisms.

## 2 Literature search and evidence selection

This article is positioned as an evidence-oriented narrative review. To enhance the transparency of the review process, targeted literature searches were conducted in PubMed, Web of Science, Embase, and the Cochrane Library for relevant studies published from January 2010 to January 2026. The search terms included “cardiac surgery,” “cardiac surgical procedures,” “perioperative sleep disturbance,” “postoperative sleep disturbance,” “sleep quality,” “sleep architecture,” “sleep latency,” “delirium,” “postoperative cognitive dysfunction,” “melatonin,” “ramelteon,” “dexmedetomidine,” “benzodiazepines,” “CBT-I,” “behavioral intervention,” “sleep hygiene,” “CPAP,” “BiPAP,” “high-flow nasal cannula,” and their combinations. For study selection, priority was given to randomized controlled trials, systematic reviews, meta-analyses, clinical guidelines, and studies directly involving cardiac surgery, perioperative care, or ICU populations. The studies included in the evidence summary tables do not represent an exhaustive list of all retrieved records. Instead, they were selected based on relevance, study design, population, outcomes, and ability to illustrate evidence strength and limitations.

When direct evidence in cardiac surgical patients was limited, relevant studies involving general surgical, ICU, chronic insomnia, or obstructive sleep apnea populations were also included where appropriate and interpreted cautiously as indirect evidence in the main text. Literature screening was primarily based on clinical relevance, methodological transparency, and the explanatory value of each study for the efficacy, safety, feasibility, and limitations of perioperative sleep management strategies.

## 3 Application of pharmacological interventions in perioperative sleep management for cardiac surgery

Different pharmacological agents may have distinct effects on subjective sleep quality, objective sleep architecture, postoperative delirium risk, and sedation depth. However, the quality of evidence and the populations to whom these interventions apply remain heterogeneous. Table 1 summarizes the key findings of representative randomized controlled trials and systematic reviews conducted in patients undergoing cardiac surgery, or in broader cohorts that included cardiac surgery subgroups. Table 2 further outlines the recommended dosage ranges, anticipated therapeutic effects, major perioperative adverse events, and essential monitoring considerations for commonly used pharmacological interventions.

**Table 1:** Summary of evidence and critical appraisal on perioperative pharmacological interventions (cardiac surgery).

Study (Author, Year)	Study Design	Population (Procedure/Sample Size)	Intervention (Dose/Timing)	Control	Primary Endpoint	Main Conclusions and Evidence Appraisal
Mehrnoush Dianatkhah, 2015 (12)	Randomized, double-blind	Elective CABG patients; *n* = 137	Melatonin 3 mg qhs; from 3 days preoperatively until discharge	Oxazepam 10 mg	Sleep quality (GSQS); delirium	Improved subjective sleep quality; the effect on delirium remains unclear, and the sample size was limited.
Han Y, 2022 (13)	Systematic review and meta-analysis	Cardiac surgery patients; total ≈ 1,714	Melatonin/Ramelteon (commonly: melatonin 1–5 mg qhs; ramelteon 8 mg qhs); from several nights preoperatively to several days postoperatively	Placebo/standard care	Incidence of POD	Meta-analysis suggested a reduced risk of POD, but intervention protocols varied substantially across studies.
H W Shin, 2024 (14)	Systematic review and meta-analysis	Various perioperative patients (including cardiac surgery)	Melatonin — various perioperative regimens (commonly: 1–5 mg PO qhs; short course pre- and/or postoperatively)	Placebo/standard care	POD; length of hospital stay	Overall evidence supported a trend toward reduced POD; however, mixed populations and publication bias limited the strength of evidence.
Stuti J Jaiswal, 2019 (15)	Randomized, double-blind, placebo-controlled	Cardiac/thoracic surgery patients requiring CPB; n ≈ 117	Ramelteon 8 mg PO qhs; initiated 1 night preoperatively, administered for up to 5–7 nights (including ICU nasogastric administration protocol)	Placebo	POD (CAM-ICU)	No significant reduction in POD was observed; sample size and event numbers limited the interpretation of effect estimates.
Alparslan Turan, 2020 (16)	Multicenter, randomized, placebo-controlled	Adult cardiac surgery patients requiring CPB; large-scale RCT	Low-dose dexmedetomidine infusion; intraoperatively to 24 h postoperatively	Placebo	Atrial fibrillation; new-onset POD	AF or POD was not reduced; hemodynamic risks such as hypotension require attention.
P Li, 2021 (17)	Systematic review and meta-analysis	Adult cardiac surgery patients; multiple RCTs	Dexmedetomidine — various perioperative regimens (e.g., short nocturnal course or continuous intraoperative infusion)	Placebo/standard sedation	POD	Pooled analysis tended to show a reduction in POD, but heterogeneity was substantial.
T Gargadennec, 2022 (18)	Multicenter, randomized, double-blind, placebo-controlled	Cardiac surgery patients ≥ 65 years; *n* = 348	Nocturnal low-dose dexmedetomidine infusion (20:00–08:00)	Placebo	POD	This was a trial protocol and therefore could not yet provide efficacy conclusions.
X Hu, 2022 (19)	Prospective observational study	Small surgical/cardiac-related cohorts (e.g., postoperative infective endocarditis patients)	Zolpidem 5–10 mg PO qhs (reduced to 5 mg in elderly); single preoperative dose or short postoperative course (1–3 nights)	Placebo/standard care	Subjective sleep score	Short-term improvement in subjective sleep was observed; the observational design limited causal inference.

CABG, coronary artery bypass grafting; qhs, every night at bedtime; GSQS, groningen sleep quality scale; POD, postoperative delirium; CPB, cardiopulmonary bypass; CAM-ICU, confusion assessment method for the intensive care unit.

**Table 2:** Common perioperative sleep-related medications: dosing, administration, and safety considerations.

Medication	Usual Initial Dose/Route	Main Mechanism/Perioperative Indications	Major Adverse Events/Precautions	Key Perioperative Monitoring Consideration	Clinical Recommendations (Perioperative Use)
Melatonin (20)	1–5 mg PO qhs; initiated 1–3 nights preoperatively and continued for 3–7 days postoperatively	Regulation of circadian rhythm; improvement of subjective sleep; potential reduction of POD risk	Generally well tolerated; occasional headache, nausea, daytime somnolence; caution in hepatic impairment	Sleep assessment (PSQI/sleep diary); delirium screening (CAM-ICU/CAM); monitoring for daytime somnolence	It may serve as a low-risk adjunctive option for non-sedative sleep management in selected high-risk patients.
Ramelteon (15)	8 mg PO qhs (commonly used)	MT1/MT2 receptor agonist; circadian rhythm modulation; evaluated for delirium prevention	Favorable safety profile; occasional somnolence or headache; absorption considerations with nasogastric administration	Sleep assessment; delirium screening; attention to absorption when administered via nasogastric tube	Limited evidence; not recommended for routine prophylactic use in cardiac surgery patients (may be considered in research settings or individualized cases)
Dexmedetomidine (16)	Intravenous infusion 0.1–0.7 μg/kg·h (protocol-dependent)	NREM sleep–like sedation; potential POD reduction (timing-dependent)	Hypotension, bradycardia; may exacerbate hemodynamic instability	Continuous vital signs (BP/HR); RASS assessment; arterial blood gas/electrolytes if indicated	Use with caution and individualization in cardiac surgery populations; contraindicated or dose-reduced in hemodynamically unstable patients; may be considered for short nocturnal sedation under monitored or research conditions
Benzodiazepines (e.g., midazolam, temazepam) (21)	Preoperative single dose: midazolam 7.5–15 mg PO; temazepam 7.5–15 mg PO qhs	Rapid sedation/hypnosis (short-term use)	Cognitive suppression, delayed emergence, respiratory depression, increased risk of falls and delirium (especially in the elderly)	Respiratory monitoring (SpO₂); level of consciousness; delirium screening	Avoid routine maintenance use perioperatively; if used short-term for severe preoperative anxiety, apply caution with enhanced monitoring
Z-drugs (e.g., zolpidem) (22)	Zolpidem 5–10 mg PO qhs (reduce by half in elderly)	Short-term improvement in sleep initiation and subjective sleep quality	Daytime somnolence, falls, delirium; increased risk with long-term or high-dose use	Assessment of daytime sedation/fall risk; delirium screening; liver function tests if indicated	May be used short-term in selected patients; avoid or use with extreme caution in elderly patients, those with OSA, or those at risk of respiratory depression
Suvorexant (orexin receptor antagonist) (23)	10–20 mg PO qhs (commonly 15 mg)	Promotes sleep maintenance; evidence suggests potential benefit in elderly postoperative patients	Daytime somnolence; rare memory impairment; caution in patients with impaired respiratory function	Sleep assessment; respiratory monitoring in patients with underlying respiratory disease	Potential alternative for elderly patients with sleep-maintenance insomnia; respiratory risk must be evaluated
Trazodone (24)	25–50 mg PO qhs	Sleep promotion with sedative and antidepressant effects (short-term use)	Hypotension, orthostatic hypotension; rare QT prolongation; excessive sedation	Blood pressure monitoring (initial dose/dose adjustment phase); ECG in patients with cardiac disease	Use cautiously in high-risk cardiac surgery patients; may be considered short-term in low-risk non-cardiac patients with monitoring
Antipsychotics (symptomatic use; e.g., haloperidol, quetiapine) (25)	Haloperidol 0.5–2 mg IV/PO PRN; quetiapine starting at 25–50 mg PO qhs	Primarily for established delirium (agitation or threat to safety)	QT prolongation (particularly with haloperidol), extrapyramidal symptoms, somnolence	Baseline and follow-up ECG (QTc); monitoring of delirium severity and extrapyramidal adverse effects	Not recommended for routine prophylaxis; use lowest effective dose for short-term symptomatic management with strict monitoring and adherence to institutional guidelines

### 3.1 Melatonin and melatonin receptor agonists

Melatonin is an essential endogenous hormone secreted by the pineal gland that plays a pivotal role in regulating circadian rhythms and maintaining the sleep–wake cycle, and aberrant melatonin secretion is closely associated with the development of sleep disturbances (26). Melatonin and its receptor agonists mainly act on brain regions such as the suprachiasmatic nucleus (SCN) through MT1 and MT2 receptors, thereby contributing to circadian rhythm synchronization and enhancing nocturnal sleep drive. Therefore, they may have theoretical value in the management of PSD related to circadian rhythm disruption (27). Perioperative melatonin supplementation is mainly used during the preoperative and early postoperative periods. It may serve as a potential adjunctive option for patients at high risk of delirium or those who are not suitable candidates for conventional hypnotic agents (13). Relevant systematic reviews and meta-analyses suggest that melatonin and melatonin receptor agonists may improve sleep quality, increase subjective sleep satisfaction, and potentially reduce the incidence of delirium in perioperative patients (13, 28). Some studies further suggest that melatonin may shorten the length of hospital stay; however, its effects on objective sleep architecture, duration of hospitalization, and delirium duration remain inconsistent and controversial (13, 29). This inconsistency may be related to differences in study populations, surgical procedures, melatonin dosage, timing of treatment initiation, treatment duration, and outcome selection. Therefore, the optimal strategy and long-term safety profile of melatonin and melatonin receptor agonists in the perioperative management of cardiac surgery patients require further clarification through large-scale, multicenter clinical investigations.

### 3.2 *α*₂-adrenergic receptor agonists

*α*₂-Adrenergic receptor agonists are a class of agents that exert their effects through highly selective activation of central *α*₂ receptors, among which dexmedetomidine is the prototypical representative, characterized by sedative, analgesic, anxiolytic, and sympatholytic properties (30). Dexmedetomidine produces sedation primarily by inhibiting noradrenergic neuronal activity in the locus coeruleus, acting on presynaptic and postsynaptic *α*₂ receptors within this nucleus to suppress arousal signaling and thereby induce a sedative state with some features resembling natural sleep (30, 31). Electroencephalographic studies have demonstrated that dexmedetomidine-induced sedation can simulate non–rapid eye movement (NREM) sleep, particularly the electroencephalographic patterns of NREM stages 2 and 3, with a significant increase in *δ*-frequency power and slow-wave activity highly analogous to physiological sleep (32, 33). Furthermore, functional magnetic resonance imaging and large-scale electroencephalographic analyses have confirmed that deep sedation induced by dexmedetomidine not only preserves rapid orienting responses to external stimuli but also exhibits thalamocortical connectivity patterns within principal arousal networks that closely resemble those observed during physiological NREM sleep (34). In the perioperative setting of cardiac surgery, dexmedetomidine has been extensively used as an adjunct sedative and is distinguished by its ability to approximate physiological sleep while posing a relatively low risk of respiratory depression, rendering it particularly suitable for patients in whom excessive sedation should be avoided, respiratory function is relatively preserved, or neurocognitive protection is desired (30, 31). In the intensive care unit (ICU), dexmedetomidine has emerged as a first-line non-benzodiazepine sedative, facilitating sleep during both mechanical ventilation and spontaneous breathing, improving sleep efficiency and objective sleep architecture without significantly affecting respiratory rate or oxygen saturation (35, 36). It should be emphasized that pharmacological sedation is not equivalent to natural sleep, even when drug-induced unconsciousness shows sleep-like electroencephalographic features (33, 37). Several clinical trials and systematic reviews suggest that dexmedetomidine may reduce the incidence of postoperative delirium in cardiac surgery patients, yet the causal relationship between sleep architecture improvement and delirium reduction remains debated, with therapeutic benefits influenced by dosage, timing of administration, and patient-specific characteristics (17, 38, 39). Current evidence shows that the findings of large randomized controlled trials and some meta-analyses are not fully consistent, suggesting that the clinical benefits of dexmedetomidine may depend on factors such as the treatment window, infusion strategy, dose range, perioperative sedation protocol, and baseline risk of delirium (16, 17). In clinical practice, the sedative and sleep-optimizing effects of dexmedetomidine must be carefully balanced against its hemodynamic adverse effects, with strict monitoring of heart rate and blood pressure to ensure safety (30, 40). Bradycardia and hypotension are more likely to occur in cases of excessive dosing or in high-risk populations, and adjustment of sedation depth using electroencephalographic or behavioral monitoring may help optimize its therapeutic profile (31, 40).

### 3.3 GABAergic hypnotic agents

GABAergic hypnotic agents primarily include benzodiazepines and non-benzodiazepine sedative-hypnotics; as central nervous system depressants, they exert their pharmacological effects through positive allosteric modulation of the *γ*-aminobutyric acid type A (GABA-A) receptor, thereby enhancing inhibitory neurotransmission in the cerebral cortex, thalamus, and other brain regions, reducing arousal levels and autonomic activity, and ultimately producing sedative and hypnotic effects (41, 42). Benzodiazepines have been shown to bind to specific sites on the GABA_A receptor complex, directly modulating chloride ion channel conductance and amplifying inhibitory signaling (41). Non-benzodiazepine hypnotics act by binding to benzodiazepine receptor sites to produce similar effects, potentiating GABAergic activity, and at higher doses their pharmacodynamic mechanisms closely resemble those of benzodiazepines (43). In the perioperative setting, although GABAergic hypnotics may shorten sleep latency and improve subjective sleep quality, they can disrupt normal sleep architecture; prolonged or high-dose use has been associated with reductions in slow-wave sleep, an increase in lighter sleep stages, and a potential elevation in the risk of perioperative cognitive dysfunction and delirium (44, 45). Furthermore, these agents may suppress respiration, induce or worsen sleep-related breathing events, and increase the risk of respiratory depression or mortality, especially when combined with opioids (41, 46). Perioperative administration of GABAergic hypnotics, whether alone or in combination, has been associated with prolonged mechanical ventilation, increased incidence of intensive care unit–acquired weakness and delirium, and a close relationship between sustained use and elevated mortality (10, 47). Some studies suggest that while benzodiazepines may provide short-term improvement in sleep disturbances, they may adversely affect cognitive function, and long-term or continuous use has been correlated with increased rates of cognitive impairment and delirium (44, 45, 48). In light of these risks, the use of GABAergic hypnotic agents for sleep promotion during the perioperative period of cardiac surgery should be carefully individualized, with rigorous consideration of the patient's clinical indications, underlying pathophysiology, and overall risk profile.

### 3.4 Effects of Non–sleep-indicated medications on sleep architecture

#### 3.4.1 Analgesics and their impact on sleep

In the perioperative period of cardiac surgery, the primary indication for analgesics is pain control rather than the direct improvement of sleep quality (11). Because surgery-related severe pain substantially disrupts postoperative sleep architecture, effective analgesia becomes a critical indirect determinant of perioperative sleep quality (49, 50). Opioids, which constitute the cornerstone of postoperative analgesia, are effective in alleviating acute pain; however, evidence indicates that they significantly interfere with sleep architecture, manifested by reductions in rapid eye movement (REM) sleep and slow-wave sleep, increased nocturnal awakenings, and a potential risk of respiratory depression (8, 49). Nonsteroidal anti-inflammatory drugs (NSAIDs) are commonly incorporated into multimodal analgesic regimens and, by attenuating inflammatory responses and pain, may contribute to improved sleep continuity; nevertheless, their sleep-promoting effect is indirect and relies on restoration of sleep architecture secondary to inflammation control (51). Different analgesic combinations exert distinct effects on sleep quality: opioids may exacerbate sleep architecture disruption, whereas NSAIDs may mitigate sleep-related adverse effects by reducing opioid requirements (11, 51). It should be emphasized that analgesic-induced sedation or mood improvement does not equate to genuine enhancement of sleep quality, and these effects should be carefully considered as important confounding factors in both research and clinical management (49).

#### 3.4.2 Effects of psychotropic medications on perioperative sleep

##### 3.4.2.1 Antipsychotics

Antipsychotics are primarily used in the perioperative setting of cardiac surgery for the management of psychiatric symptoms, such as delirium, severe behavioral disturbances, or psychotic manifestations, and are generally not administered as routine hypnotic agents; their use is typically limited to short-term treatment in the presence of clear indications, with prompt discontinuation once symptoms resolve (52, 53). Antipsychotic medications may prolong total sleep time and reduce the frequency of nocturnal awakenings; however, they can also induce alterations in sleep architecture, including an increase in non–rapid eye movement sleep and a reduction in the proportion of slow-wave sleep, and certain agents exhibit pronounced sedative properties (54). Importantly, antipsychotics may precipitate extrapyramidal symptoms and cardiovascular adverse events, including QT interval prolongation and arrhythmias, necessitating vigilant electrocardiographic monitoring and comprehensive risk assessment during the perioperative period (55, 56). Accordingly, antipsychotics should not be considered routine agents for perioperative sleep promotion and should be administered only on a short-term, individualized basis when clear psychiatric indications and safety concerns are present (53).

##### 3.4.2.2 Antidepressants

In the perioperative management of cardiac surgery, antidepressants are primarily prescribed for patients with comorbid mood disorders, and in most cases represent the continuation of preexisting pharmacotherapy initiated prior to surgery (57, 58). This strategy, aimed at maintaining emotional stability and reducing the risk of perioperative relapse of psychiatric conditions, has been identified as a critical component in multiple expert consensus statements and clinical recommendations (57, 59). Antidepressant medications exert significant effects on sleep architecture, most notably characterized by a reduction or delay in rapid eye movement (REM) sleep (60, 61). Evidence indicates that nearly all classes of antidepressants suppress REM sleep and may additionally impair sleep continuity and disrupt non–rapid eye movement sleep (61, 62). Consequently, the continuation of antidepressants during the perioperative period requires a careful balance between preserving emotional stability and mitigating potential alterations in sleep architecture (57, 63). Abrupt discontinuation is contraindicated, as it may precipitate withdrawal reactions and mood destabilization, including insomnia, dizziness, and affective disturbances, with symptoms potentially persisting from several days to months (64, 65).

## 4 Behavioral interventions in perioperative sleep management for cardiac surgery

PSD in patients undergoing cardiac surgery is multifactorial in origin, involving postoperative pain, environmental stimuli in the intensive care unit (ICU), circadian rhythm disruption, and psychological stress. Therefore, non-pharmacological interventions play a pivotal role in comprehensive management strategies. Table 3 summarizes and compares the major behavioral and environmental interventions—including cognitive behavioral therapy for insomnia (CBT-I), digital CBT-I, sleep hygiene education, earplugs and eye masks, light therapy, relaxation training, and multimodal bundles—with respect to their level of evidence, optimal implementation window, resource requirements, and inherent limitations. This comparison is intended to facilitate strategic decision-making in the development of clinical care pathways and research design.

**Table 3:** Evidence and implementation considerations for behavioral and environmental interventions.

Intervention Category	Primary Benefits for Sleep/Delirium	Implementation Window and Key Operational Points	Resources/Adherence Considerations	Limitations and Precautions
CBT-I (traditional face-to-face or nurse-led brief modified programs) (66)	Significant improvement in subjective sleep; reduction of preoperative anxiety	Optimal: 4–8 weeks preoperatively; short-course: 1–2 weeks preoperatively (3–7 sessions)	Requires trained personnel (nurses/psychologists) or outsourced platforms; adherence limited by available preoperative time window	Limited by the preoperative time window; its value and optimal protocol during the ICU phase require further clarification.
Digital CBT-I (app-based or guided online programs) (67)	Improves subjective sleep; scalable and widely accessible	Initiate ≥1–2 weeks preoperatively; backend monitoring of adherence	Requires platform licensing and IT support; low technical threshold for most patients	Accessibility, adherence, and privacy compliance in perioperative and ICU settings still need further evaluation.
Sleep hygiene education (basic sleep education) (68)	Improves sleep-related behaviors (foundational intervention)	Delivered during preoperative clinic visit or upon admission (10–15 min session)	Nurse-led; low cost; easily scalable	Highly feasible, but the effect of standalone intervention is limited.
Earplugs plus eye mask (69)	Improves subjective sleep quality; reduces nighttime awakenings	Initiate upon admission; first 72 h postoperatively are critical	Very low material cost; requires nursing supervision for adherence	Specific evidence in the perioperative setting of cardiac surgery is limited; wearing tolerance and safety monitoring should be considered.
Noise and light control (quiet hours/circadian management) (70)	Reduces awakenings; facilitates circadian rhythm recovery	Continuous implementation (particularly important during first 72 h); establishment of designated quiet hours	Requires cross-department coordination (nursing/engineering); institutional policy support	Effectiveness may be influenced by nursing procedures, ward environment, and consistency of implementation.
Music therapy (relaxation or bedtime music) (71)	Reduces sleep latency; alleviates anxiety	20–60 min before bedtime; based on patient preference	Low equipment and music library cost; generally well accepted	Effects may vary depending on music type, patient preference, and intervention duration.
Bright Light Therapy (BLT)/circadian rhythm entrainment (72)	Enhances daytime alertness; supports circadian rhythm restoration	Daytime exposure (08:00–12:00) for 30–60 min; or maximization of natural daylight	Requires portable light therapy devices or ward environmental modification	Direct evidence on sleep outcomes is limited; caution is needed in patients with eye diseases or photosensitivity.
Relaxation training/guided meditation/breathing exercises (73)	Reduces anxiety; short-term improvement in sleep initiation	10–20 min before sleep, pre- or postoperatively	Low-cost audio recordings/scripts; feasible for nursing implementation	Long-term effects and the optimal implementation strategy in the perioperative setting of cardiac surgery remain to be clarified.

### 4.1 Comprehensive cognitive behavioral interventions

Cognitive behavioral interventions constitute the theoretical foundation for the treatment of sleep disorders, emphasizing cognitive restructuring and behavioral regulation to reduce conditioned arousal responses and restore homeostatic sleep drive (74). Patients experiencing postoperative insomnia after cardiac surgery frequently exhibit maladaptive sleep-related cognitions, which exacerbate anxiety and depression and perpetuate central hyperarousal, thereby serving as a key mechanism underlying the persistence of sleep disturbances (75). Cognitive behavioral therapy, particularly CBT-I, alleviates depressive symptoms and significantly improves sleep quality and insomnia severity by identifying and correcting dysfunctional beliefs about sleep, with benefits that may persist for several months postoperatively (66). At the behavioral level, sleep restriction therapy, stimulus control therapy, and structured sleep–wake scheduling have been shown to significantly reduce sleep latency and enhance sleep efficiency, forming the core components of CBT-I (76). However, the implementation of traditional long-term CBT-I protocols in the perioperative context of cardiac surgery is constrained by practical limitations, including limited preoperative preparation time and patients’ difficulty in completing multiweek programs, which pose substantial barriers to feasibility (77). Current randomized controlled trials support CBT-I for chronic insomnia, but evidence in high-risk perioperative cardiac surgery patients remains limited (78). In addition, the short perioperative time window, postoperative pain, sedative status, ICU environmental stimulation, and reduced patient cooperation may all limit the complete implementation of traditional CBT-I in cardiac surgical patients and restrict the generalizability of its effects.

Therefore, in the perioperative cardiac surgery setting, CBT-I may be incorporated into a comprehensive management framework as a low-risk adjunctive intervention and may be particularly suitable for patients with pre-existing insomnia or sleep–wake rhythm disturbances before surgery. Its optimal implementation window, intervention intensity, and effects on postoperative sleep and neurocognitive outcomes require further validation.

### 4.2 Sleep hygiene and environmental management

#### 4.2.1 Sleep hygiene education

Sleep hygiene education aims to correct maladaptive sleep habits through structured behavioral guidance and represents a fundamental nonpharmacological intervention in the perioperative management of cardiac surgery patients (79). Its core components include avoiding bedtime caffeine, reducing evening electronic device use, maintaining regular sleep–wake schedules, and optimizing the sleep environment (79, 80). Mechanistically, sleep hygiene education reduces central arousal levels and enhances sleep continuity by limiting stimulating substances and pre-bedtime exposure to electronic media (80, 81). In clinical practice for cardiac surgery, sleep hygiene education is commonly integrated into preoperative counseling and routine nursing care; owing to its simplicity, low cost, and ease of implementation, it can be readily incorporated into comprehensive perioperative management pathways (79). It should be noted, however, that sleep hygiene education alone confers limited benefit in improving perioperative sleep quality and typically requires combination with other behavioral or pharmacological interventions to achieve optimal outcomes (82).

#### 4.2.2 ICU environmental optimization

The intensive care unit (ICU) environment can substantially disrupt sleep architecture in cardiac surgical patients, manifesting as increased nocturnal awakenings, circadian rhythm disturbances, and impaired sleep continuity (83). Continuous exposure to artificial lighting, alarm-related noise from medical equipment, and irregularly timed nursing activities directly interfere with the synchronization of central and peripheral circadian systems, resulting in deterioration of sleep quality and an increased risk of cardiovascular complications (83, 84). To address these disruptive factors, ICU environmental optimization strategies emphasize reducing nocturnal light intensity, systematically controlling or attenuating medical device noise, rationally scheduling nursing interventions within designated time windows, and employing earplugs and eye masks to minimize exogenous sensory stimulation (84–86). Following implementation of these measures, some studies have suggested improvements in subjective sleep quality, shortened duration of delirium, and modest reductions in ambient noise levels (84, 86, 87). By decreasing nocturnal arousal stimuli and strengthening circadian signaling, ICU environmental optimization may facilitate partial restoration of sleep in selected patients; however, its effectiveness is influenced by multiple confounding factors, and heterogeneity in sleep monitoring tools and outcome measures across studies contributes to ongoing uncertainty regarding the magnitude of benefit (88). In addition, measures such as noise reduction, light regulation, clustering of nursing procedures, earplugs, and eye masks are often implemented as combined interventions. This makes it difficult to distinguish the independent effect of each individual measure across studies and represents an important source of heterogeneity. Therefore, ICU environmental interventions should be regarded as an essential component of a comprehensive perioperative sleep management framework and implemented synergistically with other therapeutic modalities to achieve multidimensional optimization of sleep quality.

### 4.3 Relaxation and mind–body regulation techniques

Relaxation training, meditation therapy, breathing regulation, and mindfulness-based interventions primarily improve perioperative sleep management by suppressing excessive sympathetic nervous system activation, enhancing parasympathetic tone, regulating autonomic nervous system function, and reducing physiological and psychological arousal levels, thereby alleviating stress responses and improving sleep quality (89, 90). Patients undergoing cardiac surgery typically experience high levels of preoperative psychological stress and are therefore more susceptible to anxiety and sleep disturbances; mind–body regulation techniques contribute to reducing psychological burden and improving preoperative physiological status (91, 92). Some studies have reported that breathing regulation, relaxation training, and specific meditation practices can improve anxiety symptoms and subjective sleep experience, although evidence regarding their effects on objective sleep architecture remains limited (89, 92). Furthermore, the high-risk nature of cardiac surgery predisposes patients to anxiety and negative emotional states; therefore, the rational application of mind–body regulatory interventions may help alleviate preoperative anxiety and mild sleep disturbances (91, 93).

### 4.4 Adjunctive technological interventions in perioperative sleep management

#### 4.4.1 Biofeedback

Biofeedback is a technique that facilitates voluntary regulation of neural activity by providing real-time monitoring and feedback of physiological signals; its core principle involves training autonomic nervous system regulation through the monitoring of heart rate variability (HRV), respiratory rate, or electrodermal activity (94, 95). These physiological markers reflect vagal cardiac regulation, respiratory rhythm stability, and sympathetic arousal levels, all of which play important roles in autonomic balance and emotional regulation (96, 97). Continuous biofeedback training can enhance patients’ ability to consciously modulate autonomic activity, induce relaxation responses, alleviate tension, facilitate sleep initiation, and partially improve cognitive function (98, 99). In the perioperative context of cardiac surgery, biofeedback is more suitable for individuals with good cognitive comprehension and high treatment adherence; related studies indicate that the technique has few inherent contraindications, although it is not recommended for patients with acute psychosis or severe cognitive impairment (95). At present, the application of biofeedback in perioperative sleep management remains exploratory, and further high-quality clinical studies are required to validate its therapeutic efficacy and define its scope of indication.

#### 4.4.2 Digital CBT-I and other technological interventions

Digital cognitive behavioral therapy for insomnia (digital CBT-I) refers to the delivery of structured behavioral interventions through internet platforms or mobile intelligent terminals, enabling standardized and automated support for insomnia management (100). Its core functions include online sleep diary monitoring, circadian rhythm prompting, and personalized feedback mechanisms, which facilitate continuous tracking of patient sleep performance and provide a foundation for dynamic treatment adjustment (101). A distinctive advantage of digital CBT-I is its ability to overcome spatial and temporal limitations associated with traditional face-to-face therapy, allowing patients to receive sustained intervention during preoperative preparation and post-discharge recovery phases, thereby aligning with the multi-timepoint sleep management requirements of perioperative cardiac surgery care (101, 102). However, automated intervention algorithms currently demonstrate limited capability in accurately identifying individual dynamic clinical changes during the perioperative period; digital CBT-I remains in the early research stage, and robust large-sample evidence supporting its precision-modulation effectiveness is still insufficient (103). For ICU patients or those in the early postoperative period, fluctuations in consciousness, analgesic and sedative therapy, dependence on medical devices, and reduced cooperation with interventions may also limit the practical accessibility and completeness of digital interventions.

## 5 Respiratory support in perioperative sleep management for cardiac surgery

### 5.1 Noninvasive positive airway pressure ventilation

#### 5.1.1 Continuous positive airway pressure

Continuous positive airway pressure (CPAP) ventilation maintains upper airway patency throughout the respiratory cycle by delivering constant positive airway pressure, thereby preventing airway collapse in patients with obstructive sleep apnea (OSA) and improving ventilation by increasing the cross-sectional area of the soft palate, lateral pharyngeal walls, and tongue base airway segments (104, 105). In cardiac surgical patients, particularly those with comorbid OSA, CPAP can improve nocturnal oxygenation and may support sleep continuity by reducing apneic events and intermittent hypoxic burden. Short-term preoperative use may shorten ICU stay and duration of mechanical ventilation and improve cardiopulmonary outcomes (106, 107). However, most existing studies have focused on respiratory and clinical outcomes, and its effects on objective sleep quality and sleep architecture require further clarification. With respect to sleep architecture, CPAP reduces apnea–hypopnea events and intermittent hypoxic burden, thereby optimizing both macrostructural and microstructural sleep parameters, including reductions in the apnea–hypopnea index and nocturnal hypoxemia, which indirectly contribute to decreased cardiovascular risk and lower delirium incidence (104, 108). Furthermore, CPAP has been shown to enhance sleep spindle activity on nocturnal electroencephalography, reduce neurophysiological arousal, and provide additional objective evidence supporting improvements in sleep quality and sleep architecture (109). However, CPAP application is limited by adherence issues, including mask discomfort, device noise, air leakage, pressure intolerance, and interface-related comfort differences (110–113). Therefore, clinical application should take into account patient comfort, mask fit, air leakage, and pressure tolerance, with the interface and parameters adjusted according to actual use. In addition, inappropriate airflow or pressure settings may lead to inadequate oxygenation and increase the risk of adverse prognostic outcomes (105).

#### 5.1.2 Bilevel positive airway pressure

Bilevel positive airway pressure (BiPAP) ventilation operates by alternately delivering inspiratory positive airway pressure (IPAP) and expiratory positive airway pressure (EPAP), thereby maintaining airway patency while reducing respiratory workload (114, 115). Compared with CPAP, BiPAP provides a pressure variation pattern that is more physiologically compatible with natural breathing dynamics, particularly in clinical scenarios characterized by differential inspiratory and expiratory ventilation requirements (115, 116). The primary indications for BiPAP include obstructive sleep apnea with concomitant ventilatory dysfunction, obesity hypoventilation syndrome, and high-risk postoperative respiratory failure in cardiac surgical patients, as these populations frequently experience impaired gas exchange during the perioperative period due to reduced respiratory center sensitivity or underlying pulmonary disease, necessitating higher levels of ventilatory support to improve oxygenation and carbon dioxide elimination (117, 118). From a physiological perspective, BiPAP increases inspiratory airway pressure to augment tidal volume and reduce ventilatory workload, while lower expiratory pressure facilitates carbon dioxide clearance and alleviates diaphragmatic fatigue, thereby counteracting hypercapnia and respiratory acidosis (116, 119). Clinical evidence suggests that BiPAP improves arterial blood gas parameters and exercise tolerance in high-risk patients and alleviates atelectasis, heart failure–related respiratory compromise, and obesity-associated sleep-disordered breathing (118–120). Perioperative BiPAP application requires close monitoring of hemodynamic status and gas exchange, with preventive control of hypotension, heart rate abnormalities, and oxygenation fluctuations. Treatment parameters should be individualized, ensuring adequate mask sealing and standardized device operation to enhance patient adherence and clinical efficacy (115, 118, 121, 122). At the same time, parameter settings, interface compatibility, air leakage, and patient tolerance of pressure support may all influence real-world effectiveness. In the early postoperative period, dynamic adjustments should be made according to oxygenation status, hemodynamic changes, and patient comfort (123). Overall, owing to its adjustable pressure delivery, ventilatory support capability, and favorable physiological compatibility, BiPAP may support the management of respiratory contributors to PSD in selected high-risk cardiac surgery patients, although direct evidence on sleep-specific outcomes remains limited.

### 5.2 Oxygen therapy and high-flow respiratory support

#### 5.2.1 Conventional oxygen therapy

Conventional oxygen therapy refers to the delivery of supplemental oxygen to patients via nasal cannula or face mask to increase the oxygen concentration of inspired gas, and it represents the most widely used respiratory support modality during the perioperative period of cardiac surgery (124). The core physiological mechanism involves regulation of the partial pressure of inspired oxygen to effectively correct hypoxemia, increase alveolar and arterial oxygen tension, and optimize gas exchange (125). Oxygen undergoes a sequential partial pressure gradient from the atmosphere through the respiratory tract to the alveoli, where oxygen diffuses into pulmonary capillaries and binds to hemoglobin to form oxygenated blood, which is subsequently distributed to systemic tissues; arterial oxygen partial pressure (PaO₂) is a key parameter for evaluating oxygenation status (125, 126). Conventional oxygen therapy provides basic oxygenation support for cardiac surgical patients with mild to moderate hypoxemia, particularly during the early postoperative recovery phase (127). In clinical practice, conventional oxygen therapy is widely applied in perioperative and intensive care settings, with primary indications including active hypoxemia, early postoperative recovery, and cases in which monitoring indicators suggest borderline oxygenation status (128). Although conventional oxygen therapy can temporarily improve oxygen saturation, its sustained effect on sleep architecture and sleep quality is limited, and its airway support capacity is insufficient for patients with severe hypoxemia or concomitant sleep apnea, rendering it less effective in preventing nocturnal hypoventilation and oxygen saturation fluctuations (127, 129).

Therefore, conventional oxygen therapy is generally positioned as a basic and short-term adjunctive oxygenation strategy rather than a core therapeutic intervention. In critically ill or complex patients, more advanced respiratory support modalities, such as high-flow nasal cannula oxygen therapy or noninvasive positive pressure ventilation, are typically required to achieve superior oxygenation and sleep architecture protection.

#### 5.2.2 High-Flow nasal cannula oxygen therapy

High-flow nasal cannula oxygen therapy (HFNC) delivers heated, humidified oxygen through nasal cannulae at flow rates up to 60 L/min, providing stable oxygen concentration and airway humidification (130). The primary mechanism of HFNC involves matching or exceeding the patient's peak inspiratory flow to reduce ambient air entrainment and generate a modest positive end-expiratory pressure effect, thereby maintaining airway patency, improving lung volume, and reducing airway resistance (131). Additionally, HFNC enhances gas exchange efficiency by flushing anatomical dead space in the nasopharyngeal region with high-velocity warm and humidified oxygen, thereby reducing carbon dioxide rebreathing; this mechanism has been validated in both acute and chronic respiratory failure (132). In the perioperative context of cardiac surgery, the main indications for HFNC include postoperative hypoxemia in patients who cannot tolerate strong positive pressure ventilation, particularly in scenarios where oxygenation improvement is required after extubation but conventional oxygen delivery methods are insufficient (133–135). HFNC significantly reduces respiratory workload, improves alveolar oxygenation efficiency, and, owing to its high humidity and precise oxygen concentration control, enhances patient comfort and tolerance, thereby reducing respiratory muscle fatigue and improving post-extubation oxygenation (130, 135, 136). Randomized controlled trials and meta-analyses in cardiac surgical populations have confirmed that, compared with conventional oxygen therapy or high-flow face mask oxygenation, HFNC improves oxygenation indices, reduces the need for noninvasive ventilation, and significantly enhances patient subjective satisfaction (134, 135). However, in clinical practice, the effects of HFNC should be comprehensively evaluated in relation to patients’ flow tolerance, humidification effectiveness, oxygenation response, and device availability. In addition, the current evidence base for HFNC is largely centered on respiratory-related endpoints, such as oxygenation parameters, the need for post-extubation respiratory support, and patient comfort, with limited direct assessment of sleep architecture, nocturnal awakenings, or objective sleep quality (137). Therefore, the potential benefits of HFNC for perioperative sleep may be largely attributable to indirect effects resulting from improved oxygenation and enhanced comfort, while its independent sleep-promoting effect still requires further validation through studies using objective sleep monitoring.

## 6 Comprehensive perioperative sleep management strategy

### 6.1 Risk stratification and sleep assessment

The core objective of perioperative sleep management in cardiac surgery is to identify high-risk patients with sleep disturbances at an early stage and implement individualized risk stratification to optimize allocation of intervention resources and ensure clinical safety (138). PSD is common after cardiac surgery, with approximately 33% of patients reporting persistent postoperative sleep impairment, and female sex, advanced age, and alcohol consumption identified as major risk factors (50, 138). The prevalence of preoperative sleep disturbance can reach 63% and is closely associated with cognitive decline, delirium, and delayed recovery (50, 139). In terms of sleep assessment, the Pittsburgh Sleep Quality Index (PSQI) is a commonly used subjective screening tool during the perioperative period of cardiac surgery and can dynamically reflect changes in sleep quality; elevated PSQI scores are associated with postoperative delirium and adverse recovery outcomes (138, 139). The STOP-Bang questionnaire demonstrates high sensitivity in screening for obstructive sleep apnea (OSA) and is suitable for rapid preoperative stratification of moderate-to-severe OSA (140, 141). The severity of OSA independently predicts complications such as prolonged intubation and new-onset atrial fibrillation; therefore, screening for OSA subtypes and combining subjective screening with objective diagnostic methods, such as polysomnography and spirometry, can facilitate precise risk identification and targeted management (142, 143). Delirium is a common neuropsychiatric complication following cardiac surgery. Poor preoperative sleep quality, advanced age, and increased disease complexity represent major risk factors; thus, early identification of high-risk patients can support the development of delirium prevention strategies (139, 144). Given that cardiac surgical patients are often elderly and frequently present with multiple comorbidities, sleep-related risk profiles exhibit marked heterogeneity, necessitating risk stratification management based on demographic characteristics and medical history to enhance intervention precision and reduce the risks of postoperative delirium and delayed recovery.

### 6.2 Integrated application of multimodal interventions

**Figure 2:**
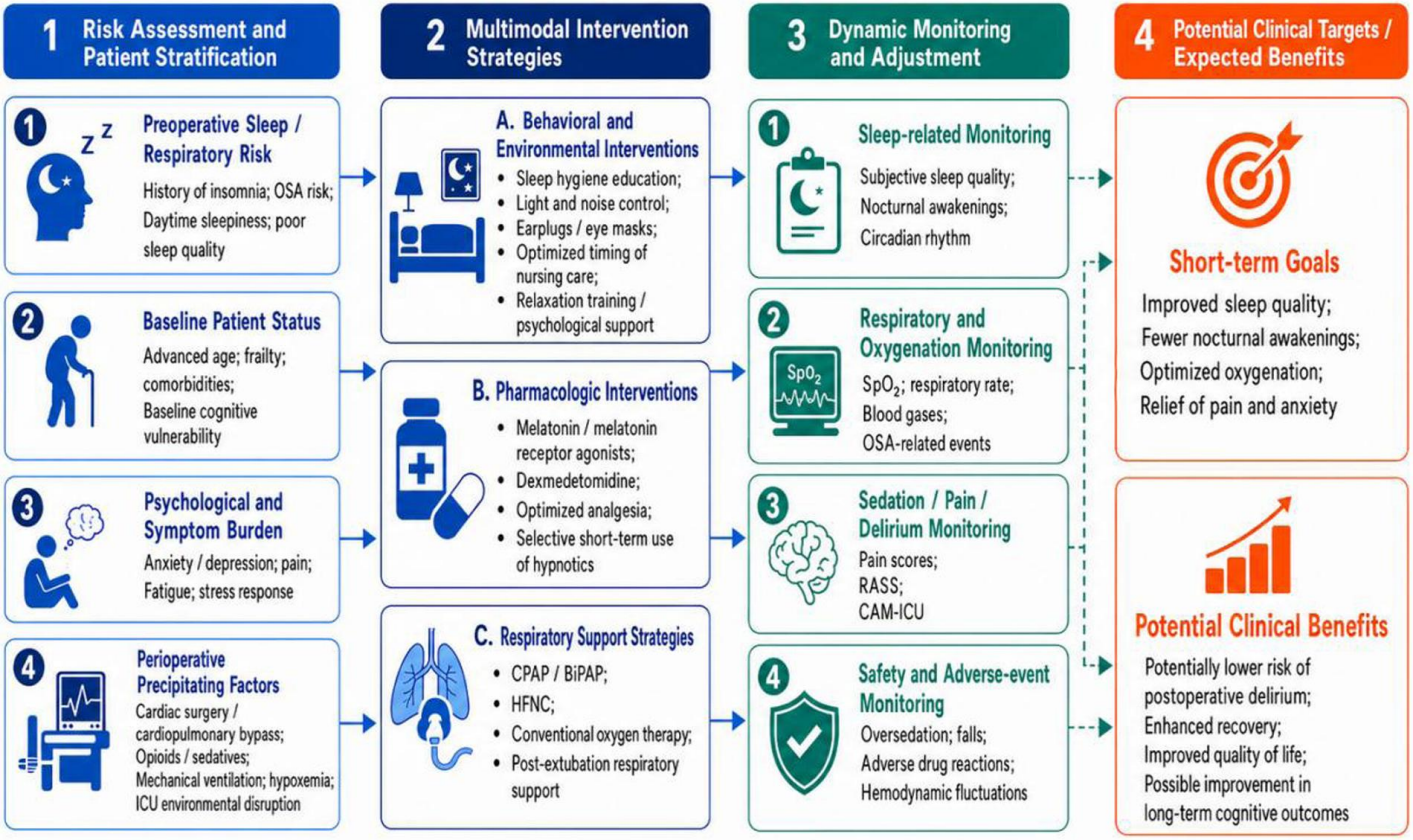
Conceptual integrated pathway for multimodal perioperative sleep management.

The concept of multimodal intervention has received increasing attention in perioperative sleep management for cardiac surgery. It emphasizes the coordinated integration of behavioral therapy, pharmacological treatment, and respiratory support (Figure 2), with the aim of establishing a comprehensive management framework based on risk stratification and clinical needs. This approach avoids overreliance on any single intervention and instead focuses on multidisciplinary collaboration and optimization across the entire perioperative pathway (145). Behavioral therapy improves sleep rhythm and reduces nocturnal awakenings through sleep hygiene education, environmental optimization, and cognitive behavioral interventions, whereas pharmacological therapy targets high-risk patients or specific etiological factors, following guideline recommendations to balance efficacy and safety while minimizing adverse reactions (50, 138). Respiratory support is primarily applied to patients with obstructive sleep apnea and postoperative hypoxemia, and improves airway patency and oxygenation through the selection of CPAP or high-flow oxygen therapy, thereby reducing the negative impact of nocturnal hypoxia on sleep architecture and cardiovascular function (146, 147). In clinical practice, individualized treatment strategies should be developed by comprehensively considering patient comorbidities, surgical complexity, age, and previous sleep history, while dynamically adjusting intervention protocols across different perioperative stages to achieve precision-oriented therapy and simultaneously prevent excessive sedation, respiratory depression, and neurocognitive impairment (138, 148). Current evidence suggests that integrated multimodal management may help improve sleep quality in patients undergoing cardiac surgery and may have potential value in reducing postoperative delirium, complications, duration of mechanical ventilation, and length of hospital stay (149, 150). However, the existing evidence is largely derived from single-intervention studies, comprehensive nursing bundles, or indirect inference, and head-to-head randomized controlled trials focusing on sleep outcomes in cardiac surgical patients remain limited. Therefore, multimodal sleep management is more appropriately regarded as a comprehensive management framework based on risk stratification, clinical rationale, and multidisciplinary collaboration. Its specific intervention combinations and implementation pathways require further standardization.

This figure summarizes a conceptual pathway incorporating risk assessment, multimodal interventions, dynamic monitoring, and potential clinical targets or expected benefits for patients undergoing cardiac surgery. It should be interpreted as an evidence-informed framework rather than a definitively proven clinical pathway.

### 6.3 Adverse event management and safety monitoring

The primary adverse events associated with perioperative sleep interventions include respiratory depression, hypoxemia, postoperative delirium, and cognitive dysfunction, all of which may significantly influence patient prognosis (151, 152). Drug-related respiratory depression is mainly associated with the central inhibitory effects of GABAergic hypnotics and opioid analgesics; patients with obstructive sleep apnea or advanced age are particularly susceptible to persistent hypoxemia, which may further precipitate reintubation, pulmonary complications, and prolonged hospitalization (151, 153). The occurrence of delirium and cognitive dysfunction is related to perioperative pharmacological interventions, intraoperative complexity, and disruption of sleep architecture; some patients may present with transient postoperative psychiatric and cognitive fluctuations, while severe cases may develop long-term cognitive impairment (152, 154). In terms of safety monitoring, key parameters include respiratory function indicators such as oxygen saturation (SpO₂) and respiratory rate, while dynamic arterial blood gas analysis and continuous pulse oximetry monitoring in intensive care settings are routinely applied in clinical practice (155). Neurocognitive status assessment tools, such as the Confusion Assessment Method for the ICU (CAM-ICU), play an important role in the early detection of delirium and cognitive abnormalities (156). Treatment protocols should be dynamically adjusted based on monitoring results, including precise control of medication dosage, stepwise optimization of respiratory support modalities, and individualized refinement of behavioral interventions to maintain a dynamic balance between sleep management and respiratory/neurocognitive safety (151, 154). Establishing a multidimensional dynamic safety evaluation system in clinical practice is essential for preventing complications, reducing mortality, and improving overall prognosis (151).

## 7 Limitations

This review has several limitations. As a narrative review, it did not perform quantitative synthesis or a rigorous risk-of-bias assessment, and the conclusions are mainly based on an integrated summary of existing studies. The available evidence varies considerably in terms of study populations, surgical procedures, intervention timing, dosage, treatment duration, and outcome measures. In addition, some studies were conducted in general ICU patients, general surgical populations, patients with chronic insomnia, or patients with obstructive sleep apnea; therefore, extrapolation of these findings to perioperative cardiac surgical patients should be approached with caution. Some studies had small sample sizes, many were single-center studies, and potential publication bias may exist, which limits the stability and generalizability of the conclusions. Across studies, the definitions and measurement tools for sleep quality, sleep architecture, sleep latency, sedation status, and delirium were not fully consistent, further increasing the complexity of result interpretation. Evidence for behavioral interventions, respiratory support, and multimodal management remains largely derived from indirect evidence or comprehensive nursing studies. In particular, head-to-head randomized controlled trials using sleep outcomes in cardiac surgical patients as primary endpoints are still lacking. Future prospective studies with large sample sizes, multicenter designs, and standardized outcome assessments are needed to further clarify the target populations, optimal implementation pathways, and clinical benefits of different interventions.

## 8 Conclusion

PSD is highly prevalent among cardiac surgical patients and is closely associated with adverse outcomes such as postoperative delirium, cognitive decline, and delayed recovery. Although various intervention strategies demonstrate potential value in improving perioperative sleep, significant differences remain in their mechanisms of action, target populations, and safety profiles. In terms of pharmacological interventions, melatonin and melatonin receptor agonists, as well as *α*₂-adrenergic receptor agonists, show promising potential in improving circadian rhythm regulation, enhancing sleep quality, and reducing the risk of neurocognitive complications; however, their optimal dosage ranges, timing of administration, and long-term safety require further validation. Although GABAergic hypnotics may shorten sleep latency, they may disrupt normal sleep architecture and increase the risks of respiratory depression and cognitive impairment; therefore, their perioperative use should be strictly restricted to appropriate clinical indications. Behavioral interventions, including cognitive behavioral therapy, sleep hygiene education, and mind–body regulation techniques, can exert auxiliary therapeutic effects by reducing central arousal levels and alleviating psychological stress responses; however, due to the limited perioperative treatment window, standardized implementation protocols for these interventions require further optimization. Respiratory support therapies, particularly noninvasive positive airway pressure ventilation and high-flow nasal cannula oxygen therapy, may indirectly support sleep continuity by improving oxygenation and respiratory comfort, rather than through a clearly established direct effect on sleep architecture. However, caution is still warranted when extrapolating existing findings to patients undergoing cardiac surgery, particularly in perioperative and ICU settings, where applicability, adherence, and safety require further clarification. Overall, perioperative sleep management in cardiac surgery should follow precision medicine principles, emphasizing risk stratification and multimodal synergistic interventions. Future research should focus on large-scale, multicenter randomized controlled trials to further clarify the efficacy, safety, and neurocognitive protection mechanisms of various intervention strategies, thereby promoting the standardization and clinical translation of perioperative sleep management.
